# Exploration of current situation of psychotropic drugs research and development in China based on drug clinical trials

**DOI:** 10.3389/fpsyt.2025.1599038

**Published:** 2025-07-15

**Authors:** Chen Xu, Yang Zhao, Xueqin Yang, Juan Zheng, Qian Tang

**Affiliations:** Department of Pharmacy, Guangyuan Central Hospital, Guangyuan, Sichuan, China

**Keywords:** psychiatric disorders, pharmacotherapy, clinical trials, innovative drugs, drug optimization, trial design

## Abstract

**Objective:**

To understand the current status of research and development (R&D) of psychotropic drugs.

**Methods:**

Retrieved psychotropic drugs clinical trials (PDCTs) registered in China from 2019 to 2024 using the platform of *chinadrugtrials.org.cn*, and systematically analyzed the data.

**Results:**

Included four perspectives: 1) for general information, we screened 1377 PDCTs, with phase bioequivalency (BE) accounting for the majority (78.5%), covering 411 pharmaceutical companies and 212 leading institutions, and the start-up time in 2024 was significantly shortened (*P* < 0.05); 2) for indications, 11 indications were involved, with the highest number of PDCTs for depression (30.9%); 3) for drugs, 222 drugs were involved, of which 52 were innovative drugs (33 with disclosed targets), and 13 were improved new drugs with six administration routes; 4) for trial design, four exploratory designs were retrieved, including population pharmacokinetics (9), pharmacogenomics (12), biomarker detection (3), and drug combination (3).

**Conclusions:**

In recent years, clinical trials of psychotropic drugs in China have been developing. Innovative targets discovery, dosage forms/drug delivery systems optimization, and exploratory designs have the potential to break the current treatment dilemma. This study introduced the hotspots and potential development directions of psychotropic drugs R&D in China from the above aspects, providing new ideas for psychiatric treatment, drug development, and clinical trial methods.

## 1 Introduction

Psychiatric disorders have become one of the leading causes of disability in the world due to the high prevalence (1, 2). According to the World Health Organization (WHO), approximately 1 billion people worldwide suffer from psychiatric disorders, accounting for about 13.0% of the total population, with anxiety (ANX, 3.8%) and depression (DEP, 3.4%) being the most common (3). As the world’s second most population country, the lifetime prevalence of psychiatric disorders in China has risen from 1.3% in 1982 and 1.4% in 1993 to 16.6% in 2019, of which ANX (7.6%) and DEP (6.8%) being the main types (4). Worryingly, with the acceleration of urbanization process and the change of social structure, the global prevalence of psychiatric disorders still has the risk of continued rapid growth (5). Psychiatric disorders pose an increasing disease, societal, and economic burden, and have become a major health issue of common concern throughout the world.

However, in the face of such enormous and urgent demands for psychiatric disorders treatment, the clinical medication strategy update remain slowly, which is intrinsically linked to the complex nature of psychiatric disorders: (1) the etiology of psychiatric disorders is the results of the combined effects of biological, social, psychological, and environmental factors, making the mechanism complex and not fully revealed; (2) the therapeutic targets for psychiatric disorders are mainly distributed in the central nervous system (CNS), which means that drugs need to cross the blood-brain barrier (BBB) to exert their effects, and conventional administration routes may reduce efficacy while increasing side effects (6–8). Given these inherent biological challenges, despite investing huge funds and time, research and development (R&D) of drugs for psychiatric disorders has lagged far behind other disease areas over the past decades (9). Most of the psychotropic drugs currently in clinical practice still act through the mechanisms of the first- or second-generation drugs, targeting dopamine receptor (DR) or 5-hydroxytryptamine (5-HT) receptor, but due to significant individual differences and frequent side effects, patients have limited benefits (10–12). The rapid growth of prevalence and the slow progress of drug development mean that the existing psychotropic drugs have been difficult to meet the clinical needs, but also mean that there is still a lot of room for exploration in the pathological mechanism and treatment methods of psychiatric disorders. Therefore, for both the healthcare industry and pharmaceutical companies, developing psychotropic drugs with new mechanisms or optimization measures is of great significance.

Good Clinical Practice (GCP) provide evidence-based evaluation of drug efficacy and safety, and have been incorporated into many countries’ laws as the gold standard for new drugs development (13, 14). The changing trends and implementation status of clinical trials can provide significant information for the direction of new drug development, research resource allocation, and trial design. With the globalization of the pharmaceutical industries, the clinical trials field in Asia is flourishing (15). China has a large market for psychotropic drugs and promising prospects for R&D of new drugs, due to the increasing prevalence of psychiatric disorders (4, 16). Based on the above, it is an interesting topic to use clinical trials information to explore the current development status and research hot spots of drugs for psychiatric disorders in China. However, there are few related reports.

In this study, we systematically analyzed the psychiatric drug clinical trials (PDCTs) registered in China from 2019 to 2024, and evaluated research progress and development prospects through innovative targets, optimization approaches, and exploratory trial designs. By integrating these perspectives, we elucidated the latest R&D trends in China’s psychiatric drugs field, and provided the reference and decision-making basis for mechanism exploration, drug development, and trial planning.

## 2 Methods

### 2.1 Information sources and search strategy

Logged in to the *chinadrugtrials.org.cn* (http://www.chinadrugtrials.org.cn), and as of December 31, 2024, the platform has registered a total of 27902 drug clinical trials. The inclusion criteria for the trials were as follows: (1) the time range for the first announcement date is from January 1, 2019 to December 31, 2024; (2) the target indications for clinical trial drugs should include the psychiatric term(s) in the Diagnostic and Statistical Manual of Mental Disorders, Fifth Edition (DSM-V). The exclusion criteria were as follows: (1) physiological disorders related to psychological factors (such as feeding and eating disorders, sleep-wake disorders, and sexual dysfunctions); (2) partial neurodevelopmental disease (such as intellectual disabilities, communication disorder, and specific learning disorder).

There were 1481 trials that met the inclusion criteria. After excluding 91 physiological disorders related to psychological factors and 13 neurodevelopmental disease, 1377 trials were ultimately selected for further analysis.

Logged in to the Center for Drug Evaluation (CDE) of China National Medical Products Administration (NMPA) (https://www.cde.org.cn/) to confirm the registration classification of the experimental drugs.

The *chinadrugtrials.org.cn* is a platform for the registration and public disclosure of drug clinical trials in China, which is supervised by the CDE. According to the Good Clinical Practice (GCP)-2020, a Chinese law jointly issued by the NMPA and the National Health Commission (NHC), which is used to regulate the entire process of drug clinical trials applied for registration in China (https://www.gov.cn/gongbao/content/2020/content_5525106.htm), all the trials disclosed on this platform have been approved by the Ethics Committee of the leading institutions. All raw data of this study were obtained from this platform.

### 2.2 Information extraction

Information extracted covered drug name, indication, drug category, phase, trial status, scope, sponsor, leading institution, registration classification, start-up time and so on. Among them, drug categories include chemical drug, biological agent, or traditional Chinese medicine (TCM)/natural medicine; phases include I, II, III, IV, bioequivalence (BE), or others (I/II or II/III); trial status includes in-progress (not recruited, recruiting, or recruitment completed), completed, or suspended/terminated; the scope includes domestic or international multi-center clinical trials; registration classification includes innovative drugs, improved new drugs, or generic drugs;start-up time = completion date of the first informed consent - approval date by the ethics committee of the leading institution.

Some clinical trials involved more than one indication, sponsor, or leading institution, whose information was independently collected.

### 2.3 Statistical analyses

Statistical analyses and graphs were performed by SPSS 22 software and GraphPad Prism 10 software. Normality test is performed using the Shapiro-Wilk (n ≤ 50) or Kolmogorov-Smirnov (n > 50) methods. Normal distribution data is represented by mean ± SD, while non-normal distribution data is represented by median (25^th^ - 75^th^), and the *t*-test and the Mann-Whitney U test are used to calculate *P* values, respectively. *P* < 0.05 means statistically significance (two-tailed).

## 3 Results

### 3.1 General information on clinical trials

**Figure 1:**
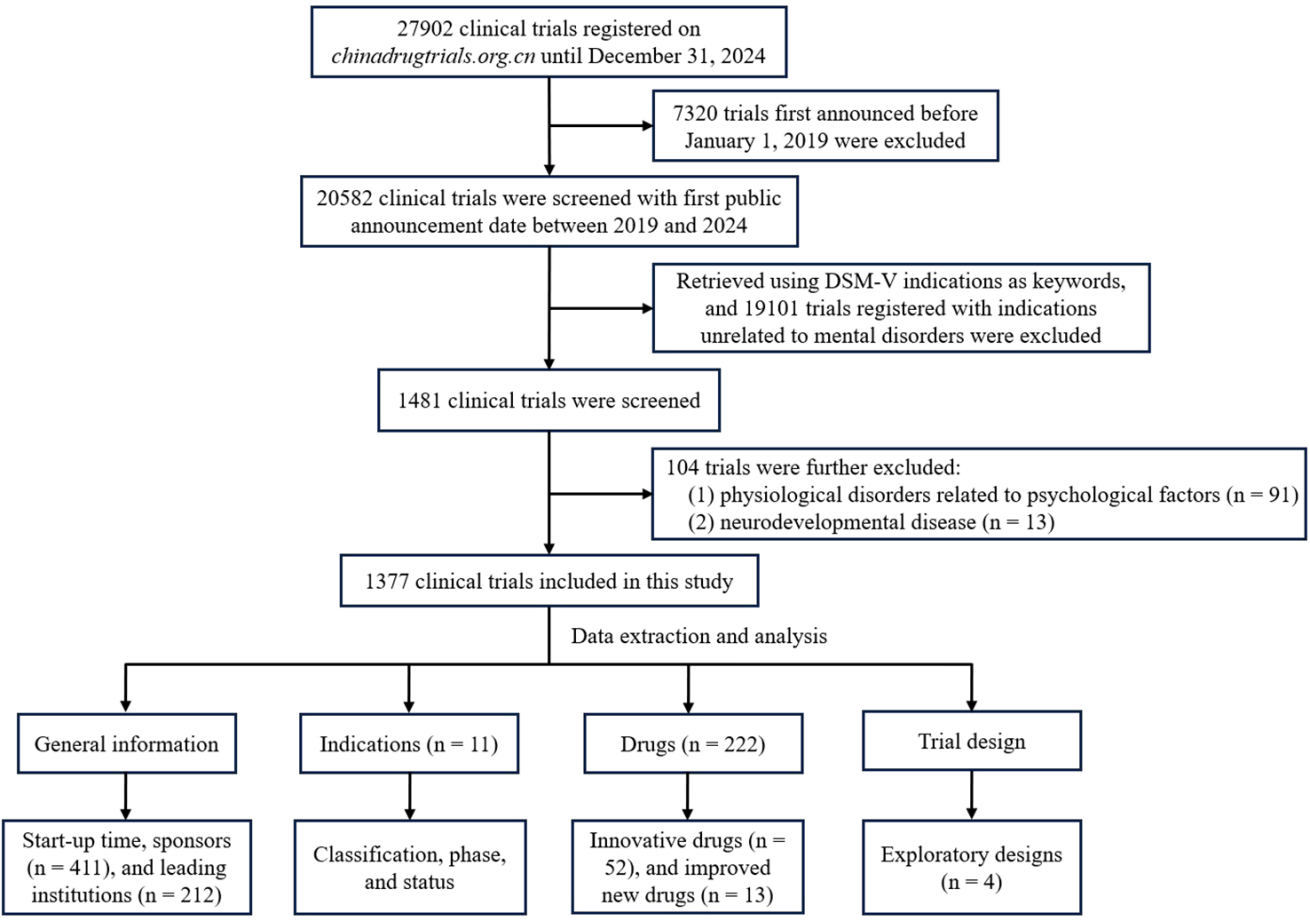
Flowchart for screening and analysis of clinical trials for psychotropic drugs.

**Figure 2:**
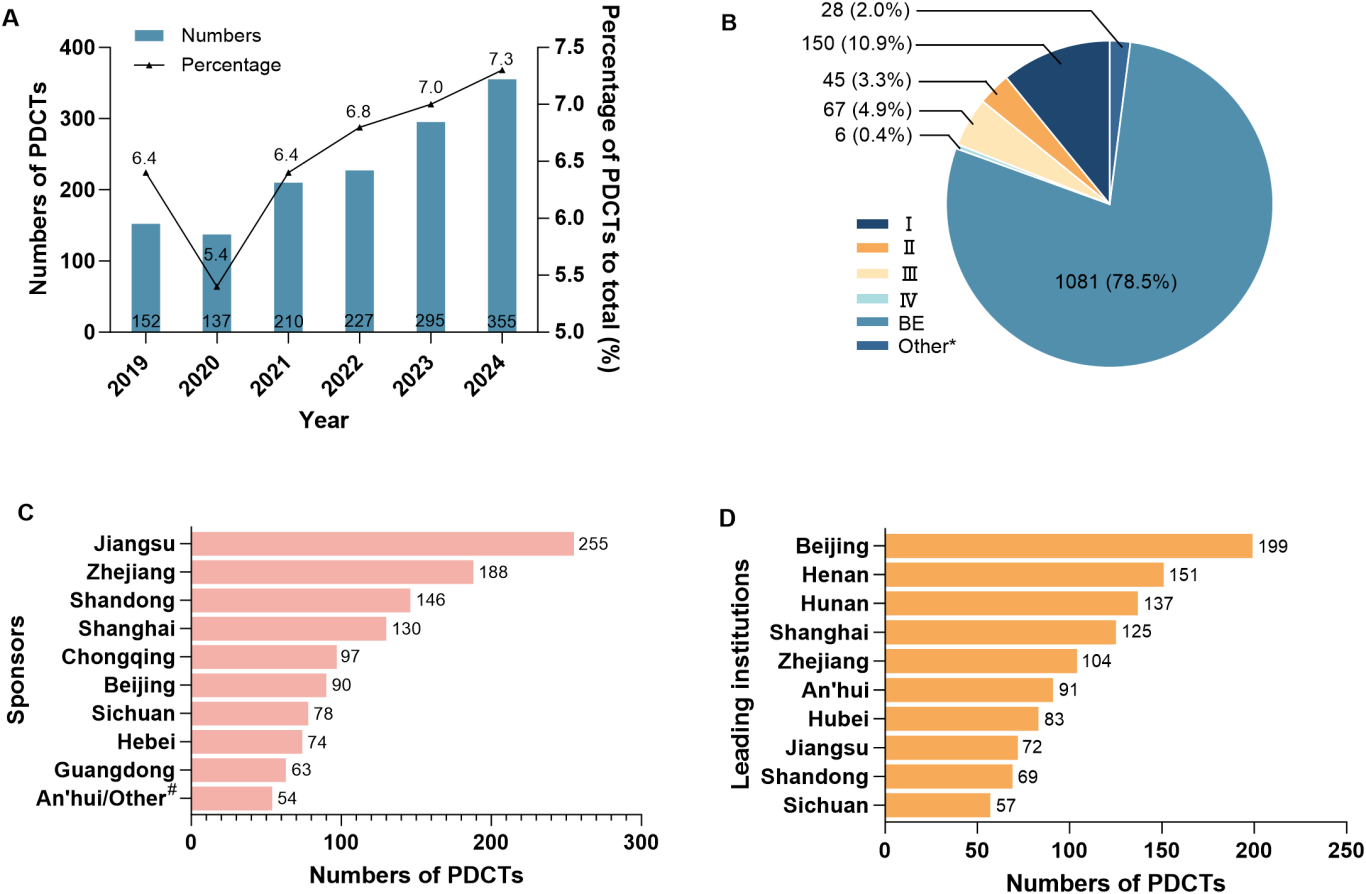
The general information on psychotropic drug clinical trials in China in 2019-2024. The registration trends **(A)** and phases **(B)** of PDCTs, as well as the geographical distribution of the top 10 sponsors **(C)** and leading institutions **(D)** in terms of registration numbers of PDCTs were displayed, respectively. A total of 1377 PDCTs were included, and some of which involved more than one sponsor or leading institution. ^*^: phase I/II or II/III; ^#^: global sponsors; PDCTs, psychiatric drug clinical trials; BE, bioequivalency.

The flowchart diagram of this study was shown (**Figure 1**). A total of 1377 PDCTs were included in the study. Except for a slightly decrease in 2020, the number of registered PDCTs and their proportion in the total number of the CDE registered trials the same year showed an increasing trend (**Figure 2A**). The trials phases were mainly BE (78.5%, **Figure 2B**). There were 1184 trials registered the first informed consent time, with a start-up time ranging from 1–693 days and an average start-up time of 80 days. Compared with 2019-2023, the start-up time for phase III, IV, BE, and other trials in 2024 was significantly shortened (**Table 1**, *P* < 0.05).

**Table 1:** The start-up time of psychiatric drug clinical trials in China in 2019-2024.

Phase	Year (days)		*P* value
	2019-2023	2024	
I, median (25^th^-75^th^)	73 (43-120)	59 (27-89)	0.157
II, median (25^th^-75^th^)	95 (62-133)	76 (21-147)	0.447
III, median (25^th^-75^th^)	156 (97-217)	55 (16-90)	**0.001**
IV, mean ± SD	153 ± 54	58^*^	**0.040**
BE, median (25^th^-75^th^)	57 (38-86)	49 (36-64)	**2.40×10^-5^**
Other, median (25^th^-75^th^)	118 (98-208)	34 (1-75)	**0.002**

There were 1184 trials registered the first informed consent time. ^*^: n = 1; BE, bioequivalency; Other: phase I/II or II/III. The mean ± SD and median (25^th^-75^th^) were used to present normal and non-normal distribution data, respectively. The One-sample *t*-test and the Mann-Whitney U test were used to calculate *P* values, respectively. Bold value indicates the statistical significance.

1346 of the 1377 clinical trials were initiated by domestic sponsors (alone or in collaboration), involving 370 domestic pharmaceutical companies distributed in 28 provinces and regions, with the highest number of the pharmaceutical companies located in Jiangsu (68). 53 trials were initiated by global sponsors (alone or in collaboration), involving 41 global pharmaceutical companies distributed in 13 countries and regions, with the highest number of pharmaceutical companies coming from the U.S. (17). There were 26 international multi-center trials, of which four were initiated by domestic sponsors alone, involving three companies located in Shanghai (2) and An’hui (1), respectively.

There were 212 leading institutions distributed in 27 provinces and regions, with the highest number of the leading institutions located in Beijing (199). Eight leading institutions undertook international multi-center trials, located in three provinces which were Beijing (5), Shanghai (2), and Shandong (1), respectively.

The geographical distribution of the top 10 sponsors (**Figure 2C**) and leading institutions (**Figure 2D**) in terms of the number of PDCTs was shown, respectively.

### 3.2 Indications

The 1377 clinical trials involved 11 indications, including 426 for DEP (30.9%), 306 for schizophrenia (SC, 22.2%), 195 for parkinsonism (PD, 14.2%), 155 for ANX(11.3%), 117 for Alzheimer’s disease (AD, 8.5%), 91 for bipolar disorder (BD, 6.6%), 51 for obsessive-compulsive disorder (OCD, 3.7%), 11 for withdrawal symptoms (WS, 0.8%), 11 for tourette syndrome (TS, 0.8%), 10 for delirium (0.7%), and four for autism (0.3%). The PDCTs information for those indications was presented (**Table 2**).

**Table 2:** Drug clinical trial information on psychiatric disorders in China in 2019-2024.

Indication	*n* ^*^	Classification			Phase						Status		
		Chemical drug	Biological agent	TCM/Natural medicine	I	II	III	IV	BE	Other	In Progress	Completed	Terminated/ Suspended
DEP	426	418	0	8	43	19	12	1	346	5	122	291	13
SC	306	306	0	0	36	5	14	1	241	9	94	199	13
PD	195	193	2	0	30	8	10	0	144	3	68	127	0
ANX	155	151	0	4	3	2	6	1	141	2	35	117	3
AD	117	97	14	6	35	10	17	2	46	7	52	57	8
BD	91	91	0	0	1	0	5	0	84	1	22	63	6
OCD	51	51	0	0	0	0	2	0	49	0	7	44	0
WS	11	10	0	1	1	0	0	0	10	0	4	7	0
TS	11	9	0	2	0	0	1	1	9	0	3	7	1
Delirium	10	10	0	0	1	0	0	0	8	1	2	8	0
Autism	4	4	0	0	0	1	0	0	3	0	1	3	0

DEP, depression; SC, schizophrenia; PD, parkinsonism; ANX, anxiety; AD, Alzheimer’s disease; BD, bipolar disorder; OCD, obsessive-compulsive disorder; WS, withdrawal symptoms; TS, tourette syndrome; TCM, traditional Chinese medicine; BE, bioequivalency; Other, phase I/II or II/III. There were a total of 11 indications. Some clinical trials involved more than one indication. *: number of clinical trial registrations.

The categories of drugs for AD clinical trials were chemical drug (82.9%), biological agent (12.0%), and TCM/natural medicine (5.1%); the categories of drugs for DEP, ANX, WS, and TS clinical trials were chemical drug (98.1%, 97.4%, 90.9%, and 81.8%, respectively) and TCM/natural medicine (1.9%, 2.6%, 9.1%, and 18.2%, respectively); the categories of drugs for PD clinical trials were chemical drug (99.0%) and biologics agent (1.0%). The category of drugs for the remaining indications was chemical drug.

Clinical trials for AD had a high proportion of phase BE (39.3%) and I (30.0%), while other indications were dominated by phase BE. The completion rate of clinical trials for OCD and delirium was high, at 86.3% and 80.0%, respectively. The terminated/suspended rate of clinical trials for TS was the highest, at 9.1%.

### 3.3 Drugs

**Figure 3:**
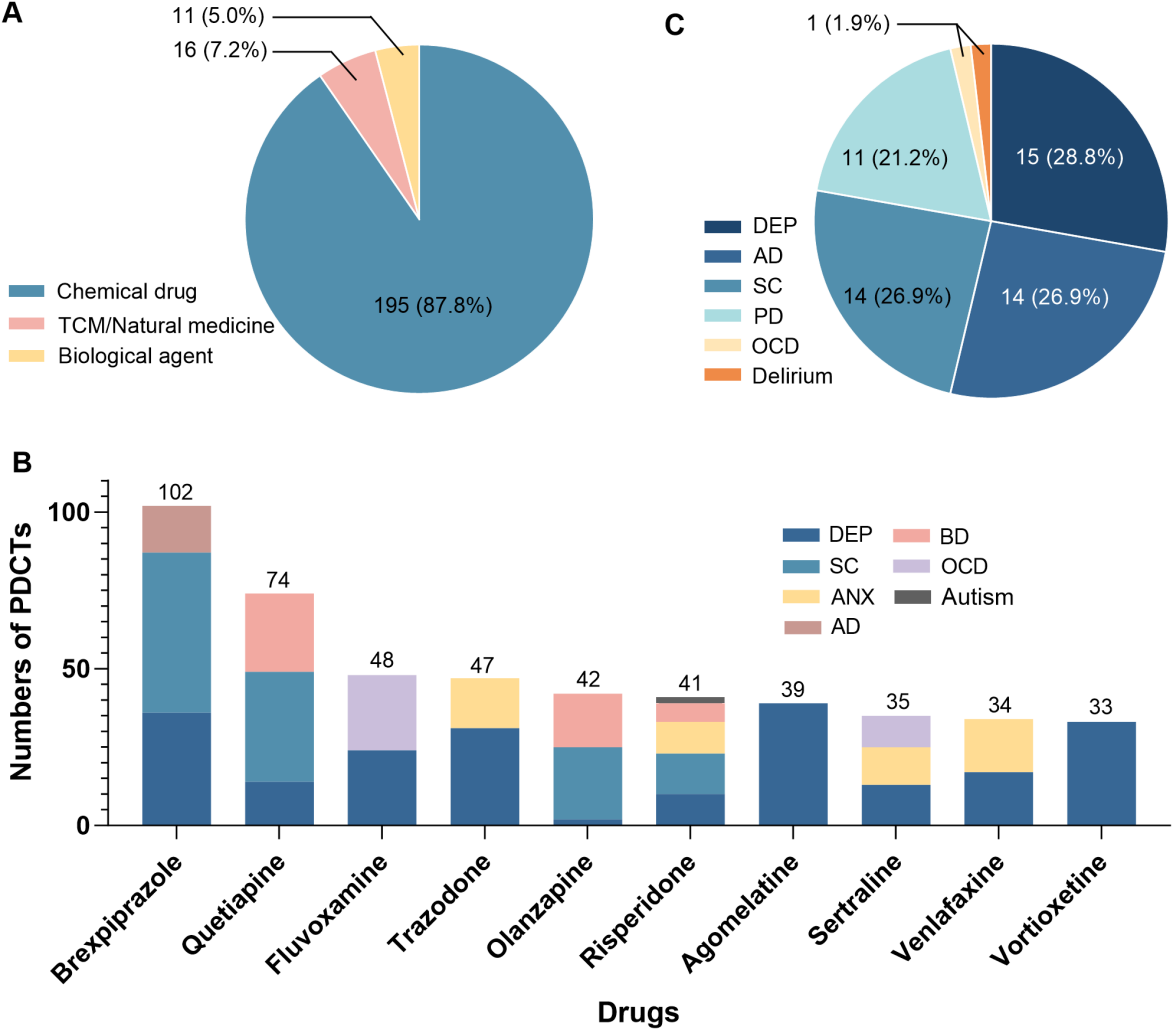
Information of drugs involved in psychiatric drug clinical trials in China in 2019-2024. The distribution of categories for all psychiatric drugs **(A)**, indication distribution for the top 10 drugs with the highest number of PDCTs **(B)**, and indication distribution for innovative psychiatric drugs **(C)** were displayed, respectively. The total number of psychiatric drugs was 222, of which 52 were innovative drugs. Some drugs participated in two or more indications. TCM, traditional Chinese medicine; DEP, depression; SC, schizophrenia; PD, parkinsonism; ANX, anxiety; AD, Alzheimer’s disease; BD, bipolar disorder; OCD, obsessive-compulsive disorder; PDCTs, psychiatric drug clinical trials.

The 1377 clinical trials involved 222 drugs, and the drug categories was shown in **Figure 3A**. The indication distribution of the top 10 drugs with the highest number of PDCTs was shown, all of which were generic drugs (**Figure 3B**).

Selected innovative drugs from the 222 drugs based on the screening criteria as follows: (1) being approved for investigational new drug application in China for the first time between 2019 and 2024; and (2) being registered as Class 1 (innovative drugs) in the CDE. The 52 (23.4%) innovative drugs were screened, participated in 97 PDCTs for six indications (some drugs participated in two or more indications). The indication with the highest number of innovative drugs was DEP (15), followed by SC (14) and AD (14) (**Figure 3C**).

There were 33 innovative drugs that publicized the targets, as basic characteristics in **Table 3**. A total of 28 targets were disclosed, which were classified into five categories based on molecular types: G protein-coupled receptors (GPCR, 53.6%), enzymes (14.3%), ion channel receptors (ICR, 7.1%), transporters (7.1%), and others (17.9%). There were 6 (18.2%) drugs targeting traditional DR and/or 5-HT receptors only, while other drugs involved novel targets. The innovative targets were mainly focused on amyloid β-protein (Aβ, 5), γ-aminobutyric acid A receptor subunit (GABA_A_R, 3), muscarinic M1/M4 receptors (mAChRM1/M4, 2), and brain derived neurotrophic factor (BDNF, 2). The proportion of drugs acting on these targets was 12/33 (36.4%).

**Table 3:** Basic characteristics of innovative drugs in psychiatric drug clinical trials in China in 2019-2024.

Drug	Target	Classification	Indication (*n* ^*^)	Phase	Drug	Target	Classification	Indication (*n*)	Phase
SIPI6398	5-HT1A;5-HT2A; DRD2; DRD3	GPCR	SC (4)	II	NORA520	GABA_A_R	ICR	DEP (1)	I
JX11502MA	5-HT1A;5-HT2A; DRD2; DRD3	GPCR	SC (2)	II	KH607	GABA_A_R	ICR	DEP (1)	I
HS-10380	5-HT2A; DRD2; DRD3	GPCR	SC (4)	II	GW201	NMDAR	ICR	DEP (1)	I
GW117	5-HT2C; MT1; MT2	GPCR	DEP (3)	II	PQ912	QPCT	Enzymes	AD (1)	II
LPM787000048	5-HT2C; TAAR1	GPCR	SC (2); AD (2)	I	WXWH0226	LRRK2	Enzymes	PD (1)	I
AM006	DR; DRIP	GPCR	PD (2)	II	HEC12 2505MsOH	MAO-B	Enzymes	PD (1)	I
NH300231	5-HT2A; DR	GPCR	SC (1)	I	MK-8189	PDE 10	Enzymes	SC (1)	I
Aticaprant	KOR	GPCR	DEP (3)	III	BI 425809	GlyT1	Transporter	SC (3)	III
KarXT	mAChRM1/M4	GPCR	SC (1); AD (1)	III	SHR-1707	Aβ	Others (amyloid protein)	AD (3)	II
VG081821AC	A2aR	GPCR	PD (2)	II	Remternetug	Aβ	Others (amyloid protein)	AD (2)	III
NH130	5-HT2A	GPCR	PD (2)	I	RP902	Aβ	Others (amyloid protein)	AD (2)	I
HS-10506	OX2R	GPCR	DEP (2)	I	BAN2401	Aβ	Others (amyloid protein)	AD (1)	III
NS-136	mAChRM4	GPCR	SC (1)	I	OAB-14	Aβ	Others (amyloid protein)	AD (1)	I
NH102	5-HT2A; SLC6A3	GPCR Transporter	DEP (2)	I	JS1-1-01	BDNF	Others (neurotrophic factors)	DEP (3)	II
Liafensine	5-HT; Dopamine; NE	GPCR Others (neurotransmitter)	DEP (1)	II	BrAD-R13	BDNF	Others (neurotrophic factors)	AD (1)	I
HS-10353	GABA_A_R	ICR	DEP (8)	II	QD202	LYZ; SYP	Others (synaptic function regulation)	AD (1)	I
MN-08	NMDAR	ICR	AD (1)	II					

There were 33 innovative drugs for psychiatric disorders that have disclosed a total of 28 targets. The targets were classified into five categories based on molecular types. ^*^: number of clinical trial registrations; 5-HT, 5-hydroxytryptamine receptor; DR, dopamine receptor; MT1, melatonin MT1; MT2, melatonin MT2; TAAR1, trace amine-associated receptor 1; DRIP, dopamine receptor interacting protein; KOR, kappa-type opioid receptor; mAChRM1/M4, muscarinic M1/M4 receptors; A2aR, adenosine A2a receptor; OX2R, orexin type 2 receptor; SLC6A3, solute carrier family 6 member 3; NE, norepinephrine; GABA_A_R, γ-aminobutyric acid A receptor subunit; NMDAR, N-methyl-D-aspartate receptor; QPCT, glutaminyl-peptide cyclotransferase; LRRK2, leucine rich repeat kinase 2; MAO-B, monoamine oxidase B; PDE 10, phosphodiesterase 10; GlyT1, glycine transporter 1; Aβ, amyloid β-protein; BDNF, brain derived neurotrophic factor; LYZ, lysozyme; SYP, synaptophysin; GPCR, G protein-coupled receptors; ICR, ion channel receptors; DEP, depression; SC, schizophrenia; AD, Alzheimer’s disease; PD, parkinsonism.

Selected improved new drugs from the 222 drugs based on the screening criteria as follows: (1) same as the criterion (1) for innovative drugs; and (2) being registered as Class 2 (improved new drugs) in the CDE. There were 13 drugs were screened out involved in 12 dosage forms and six administration routes (**Table 4**). Traditional oral and injection administration routes account for a large proportion (75%). Additionally, there are four other routes of administration, namely: intranasal, transmucosal, rectal, and transdermal.

**Table 4:** Basic characteristics of improved new drugs in psychiatric drug clinical trials in China in 2019-2024.

Drug	Indication	Phase	Dosage form	Administration Route
Olanzapine	SC/BD	BE/I	Oral dissolving film	Oral
Brexpiprazole	SC/DEP	BE/I	Oral dissolving film	Oral
BCM857	SC	I	Oral dissolving film	Oral
N2106	SC	I	Oral dissolving film	Oral
Donepezil	AD	I	Oral dissolving film	Oral
Huperzine A	AD	I	Controlled-release tablet	Oral
HRG2010	PD	III	Sustained-release capsule	Oral
TV-44749 (Olanzapine)	SC	I	Sustained-release suspension	Injection
Aripiprazole	SC	I	Sustained-release microspheres	Injection
Rotigotine	PD	I/III	Sustained-release microspheres	Injection
Brexpiprazole	SC	I	Long-acting injection	Injection
Donepezil	AD	I	Injection	Injection
(R)-Ketamine	DEP	I	Nasal spray	Intranasal
Rasagiline	PD	I	Sublingual film	Transmucosal
Midazolam	ANX	I	Gel	Rectal
Asenapine	SC	Other (pharmacokinetics)	Patch	Transdermal

There were 13 improved new drugs involved in 12 dosage forms and six administration routes. Brexpiprazole and donepezil had two dosage forms, respectively. SC, schizophrenia; BD, bipolar disorder; DEP, depression; AD, Alzheimer’s disease; PD, parkinsonism; ANX, anxiety.

### 3.4 Trial design

The 1377 clinical trials were screened for trial objective. Some of the trials had further exploratory design, including population pharmacokinetics (PopPK), pharmacogenomics (PGx), biomarker detection, and drug combination (**Table 5**).

**Table 5:** Exploratory design of psychiatric drug clinical trials in China in 2019-2024.

Objective	Indication	Registration ID	Drugs	Objective	Indication	Registration ID	Drugs
PopPK	AD	CTR20210187	GV-971	PGx	DEP	CTR20220758; CTR20222884; CTR20240090	SAL0114
		CTR20243157	50561				
		CTR20243461	HRG2010				
	ANX	CTR20231392; CTR20231405	Buagafuran			CTR20221896	Liafensine
						CTR20222150	JS1-1-01
		CTR20231959	Toludesvenlafaxine			CTR20233032	LV232
	DEP	CTR20211677; CTR20230246	Ammoxetine		SC	CTR20213168; CTR20213353	SEP-363856
	SC	CTR20213353	SEP-363856			CTR20192086	Pomaglumetad methionil
Biomarker	AD	CTR20210187	GV-971			CTR20201189	WenDanPian
	PD	CTR20243324	TJ0113			CTR20201480	Aripiprazole
	DEP	CTR20211677	Ammoxetine			CTR20231088	KarXT
Drug combination	DEP	CTR20233237; CTR20233277	Aticaprant				
		CTR20222884	SAL0114				

PopPK, population pharmacokinetics; PGx, pharmacogenetic; AD, Alzheimer’s disease; ANX, anxiety; DEP, depression; SC, schizophrenia; PD, parkinsonism.

Nine trials were designed with PopPK involved four indications which were DEP, SC, ANX, and AD.

12 trials were designed with PGx involved two indications which were DEP and SC. Among them, six trials explored the relationship between *CYP2D6* gene polymorphisms and pharmacokinetics, with registration ID was CTR20220758, CTR20222884, CTR20240090, CTR20201480, CTR20213168, and CTR20213353, respectively; one trial explored the *norepinephrine transporter protein* (*net*) *T182C* locus gene polymorphisms, with registration ID was CTR20201189.

Three trials proposed biomarker detection involved three indications which were DEP, PD, and AD. Among them, one trial explored the changes in inflammatory factors, with registration ID was CTR20243324, and the other one trial detected biomarkers of intestinal metabolites, with registration ID was CTR20210187.

Three trials were designed with drug combination, and all for DEP. Among them, two trials explored the efficacy and safety of combining Aticaprant with SSRI or SNRI therapy, with registration ID was CTR20233277 and CTR20233237, respectively; the other one trial explored the efficacy and safety of combining SAL0114 with bupropion, with registration ID was CTR20222884.

## 4 Discussion

Since the 1990s, psychiatric disorders have been recognized as one of the top 10 global burdens, with a consistently high prevalence rate that is thought to be closely related to disability and increased economic burdens (17). However, because of the complex etiology, although great progress has been made in the research on the pathogenesis of psychiatric disorders, strategies that have a significant impact on disease diagnosis and treatment have not been developed. Therefore, designing effective drug therapies for psychiatric disorders is a common challenge and urgent need worldwide, which can be reflected in our research findings that the number of PDCTs in China has shown an increasing trend over the past few years. At the same time, with the WHO announcing the end of the COVID-19 global emergency in 2023, the trial team cooperation as well as the subject recruitment has become more convenient, so we showed a significant shorten in start-up time in our study, which provides favorable conditions for the efficient implementation of PDCTs and the acceleration of the new drugs R&D progress. It is worth noting that whether in terms of the number of PDCTs or the number of innovative drugs, the vast majority of involved indications were DEP, SC, and PD from our results. It suggested that the current research still focus on common psychiatric disorders, and the development of uncommon psychiatric diagnosis and treatment is slow and needs more attention.

There are several aspects that can contribute to the success of therapeutic drugs: (1) discovery and rational design of new compounds with better efficacy and safety; (2) development of improved dosage forms and alternative drug delivery systems to increase compliance; and (3) adoption of exploratory strategies such as PopPK, genomics, combination therapy, and biomarker detection to achieve precision pharmacotherapy (18–22). This study will also discuss the current status of R&D of psychotropic drugs in China from the above aspects.

### 4.1 Innovative targets

The first- and second-generation psychotropic drugs are still the mainstream in clinical practice currently for limited R&D progress. Due to the complexity and heterogeneity of psychiatric disorders, developing effective and fewer side effects drugs has become a challenge. It is encouraging that approximately 80% of the innovative drugs with disclosed targets in our study were novel mechanisms (**Table 3**). Among them, Aβ, GABA_A_R, mAChRM1/M4, and BDNF were designed as targets by two or more drugs.

AD is a progressive neurodegenerative disorder with characteristics are memory loss, cognitive impairment, and behavioral disturbances (23). One of the mainstream views regarding the pathogenesis of AD currently is amyloid cascade hypothesis, which posits that the deposition of Aβ is a key driving factor for early disease progression in AD, and leads to subsequent pathological changes (24). Recent studies on genetics, pathology, and biomarkers have also provided evidence for this hypothesis (25–27). Therefore, targeting the reduction of Aβ levels in the brain of patients has been a foundational strategy to improve AD symptoms in the last few decades (28). In the past four years, the U.S. Food and Drug Administration (FDA) has approved three anti-Aβ monoclonal antibodies (aducanumab, lecanemab, and donanemab) consecutively, ushering in a new era of targeted therapy for AD (29–31). Among them, lecanemab (BAN2401) was approved by the FDA in 2023, becoming the first fully approved Aβ-antibody drug for AD treatment. This drug is conducting a phase III trial (CTR20200005) in China as shown in our study. From the results, 10 of 33 innovative drugs were targeted for AD, with half acting on Aβ, which suggests that the current focus of R&D for AD treatment in China is also centered on Aβ.

SC is considered as one of the most serious psychiatric disorders (32). Although drug development for SC has been ongoing for 70 years, all antipsychotics currently approved are antagonists or partial agonists at the dopamine D_2_ receptor (DRD2) (33). So, limitations of therapeutic efficacy and frequent side effects are pervasive shortcoming. In the 1990s, the mAChRsM1/M4 receptor agonist xanomeline demonstrated its potential for the first time in treating AD and SC (34, 35). The mechanism of this drug may be to activate acetylcholine signaling by stimulating mAChRs, rather than blocking DRD2 (36). However, due to the similarity in structure among the five subtypes, mAChR-targeted therapy can affect receptor subtypes distributed in different tissues, causing serious side effects and hindering further drug development (37–39). The successful development of the drug named KarXT (Cobenfy) solved this problem. It can not only selectively excite mAChRsM1/M4 receptor to exert pharmacological effects, but also inhibit muscarine receptors to suppress peripheral side effects (40, 41). KarXT was full approved by the FDA in 2024, becoming the first mAChR-targeted drug for adult SC (42). It is conducting a phase III trial (CTR20231088) in China now. In addition, a small molecule M4 selective agonist named NS-136, independently developed by China, is currently undergoing Phase 1 trials (CTR20244269).

GABA_A_R is an ICR that inhibits neural excitability by promoting chloride ion influx (43). Its signal dysregulation is related to various neuropsychiatric disorders, such as ANX, DEP, and AD (44). Brexanolone is a positive allosteric modulator of GABA_A_, which can enhance the inhibitory effects of GABA_A_. It was approved by the FDA in 2019 as the first drug for postpartum depression (45, 46). BDNF promotes neuroprotection and neuroregeneration (47). Neurodegenerative diseases and neuropsychiatric disorders may be related to insufficient neuronal supply of BDNF, and over the past 20 years, BDNF has been regarded as a key factor in the treatment of neuropsychiatric disorders (48, 49). The evidence indicating the relationship between decrease of BDNF level and the progression of PD is increasing (50, 51). Due to the lack of a cure for PD, BDNF has a promising prospect in this area. Besides, the targets mentioned in this study, such as kappa-type opioid receptor (KOR), orexin type 2 receptor (OX2R), and N-methyl-D-aspartate receptor (NMDAR) and so on, may also become new mechanisms for treating psychiatric disorders (52–54). The launch of clinical trials for these potential drugs is expected to break the decades-long treatment dilemma, heralding the arrival of a new era of non-traditional targeted therapy for psychiatric disorders.

### 4.2 Drug optimization

Many psychotropic drugs are administered via traditional oral routes, which face challenges such as first-pass effect, limited BBB penetration, low bioavailability, and systemic side effects, making it difficult to achieve optimal drug level (55). To solves these issues, several optimization strategies for existing drugs are proposed. In our research, there were some improved new drugs that adopted methods such as changing drug delivery system (DDS) and administration route to avoid BBB and eliminate first-pass effect, in order to improve the pharmacokinetic process and enhance drug efficacy.

Due to BBB, most substances in the blood are difficult to enter the brain, making it very challenging for drugs to cross the BBB distribution, especially in neurological diseases that may cause BBB dysfunction and make this process even more complex (56). So how to avoid the BBB and directly deliver drugs to the brain is an important idea to improve the distribution of psychotropic drugs in the CNS. As an alternative DDS, the mechanism of nose-to-brain delivery is that drugs are first absorbed through the nasal mucosa, then transported retrogradely along the axons of the trigeminal nerve, and ultimately direct reach the brainstem and spinal cord (57). This approach provides a non-invasive method to avoid BBB while also avoiding first-pass effect. It has advantages including rapid onset, high bioavailability, and reduced systemic exposure, making it highly valuable for clinical applications (58, 59). Ketamine is an anesthetic and analgesic drug that is commonly administered by injection (60). The S(+)-isomer named esketamine was approved for market in 1997, and received initial approval by the FDA for treatment-resistant depression in 2019 (61). The bioavailability of its nasal spray reached about 50%, which can rapidly improve DEP symptoms and provide golden-hour window intervention for patients at risk of acute suicide (62). (R)-ketamine hydrochloride is the levorotatory form of ketamine, and the phase I clinical trials (CTR20212471 and CTR20191785) of its nasal spray for treatment-resistant depression has been completed in China. However, nose-to-brain also has certain shortcomings. On the one hand, this method has limited drug loading capacity, which restricts the amount of drug that can be delivered at once; On the other hand, the nasal cavity has a self-defense function that limits the efficiency of drug delivery (63).

The traditional oral route has problems such as first-pass effect, enzyme digestion attack, and gastrointestinal irritation, etc. In addition, some patients with psychiatric disorders are accompanied by poor compliance and swallowing difficulties. Altering drug dosage form/administration route provides a conventional solution, such as injectables, transdermal delivery (gel, ointment, patch, etc.), and mucosal administration (oral, nasal, rectal, etc.), as shown in **Table 4** (64–66). Among them, transdermal delivery, as a non-invasive and self-administration method, allows drugs to be absorbed through the skin and minimizes the liver first-pass effect (67). Compared with traditional semisolid dosage forms (such as ointments and gels), transdermal patches overcome the limitations in dose accuracy and application consistency, while enabling reduced the dosing frequency through sustained drug release (68). Combining the above, patches are well suited as alternative formulations to traditional oral or injectable psychotropic drugs (69). Asenapine transdermal patch (Secuado^®^) was approved by the FDA in 2019, and it is the only FDA-approved patch for SC in adults (70). An asenapine transdermal patch clinical trial (CTR20240253) conducted in China employs sustained-release technology to extends the dosing interval, which is expected to significantly enhance patient compliance compared with Secuado^®^. However, patches also exhibit certain limitations, such as constrained drug loading capacity, requirements for drug solubility and molecular weight, and skin barrier, which restrict the improvement of some psychotropic drug formulations (71).

At present, multiple systematic technological paths are expected to break through the bottleneck of dosage forms in drug optimization schemes. With the supports of emerging technologies such as molecular structure modification, nanoformulation technologies, solvent casting systems, 3D printing, and artificial intelligence, traditional delivery routes of psychotropic drugs are expected to undergo revolutionary changes (72–74).

### 4.3 Exploratory design

There are still significant gaps in the diagnosis and treatment of psychiatric disorders that needs to be filled. Drug therapy is an important way of psychiatric disorders treatment. The efficacy and tolerability of drugs are key factors in determining whether patients can benefit from their treatment. However, due to individual differences, it usually takes several months to years to adjust the dosing regimen in clinical practice in order to find suitable therapeutic drugs (75). This has led to a series of problems such as poor disease prognosis and reduced compliance. These issues are driving the pharmaceutical industry to shift from traditional trial design to patient-centered exploratory design. The four exploratory designs in this study also suggest the occurrence of this transformation.

Distinct from traditional pharmacokinetics, PopPK utilizes a large number of clinical samples to quantify the factors contributing to drug concentration differences in the target population, in order to determine and optimize drug dosage (76, 77). Through Bayesian forecasting, PopPK analysis can be used to estimate individual pharmacokinetic parameters and customize personalized dosing regimens (78). The approved dosage strategies for many drugs used in different therapeutic fields are based on PopPK analysis. A study on PopPK of BAN2401 for AD used data from three clinical trials, including two phase I trials and one phase II trial, to identified individual differences in BAN2401 pharmacokinetic parameters, and the synergy of factors such as height and gender (79). In our study, nine trials were described as conducting PopPK. Among them, Toludesvenlafaxine, an innovative anti-anxiety drug with independent intellectual property rights in China, has been approved for market by the CDE in 2022 (80). The drug is currently undergoing a phase III trial in China to evaluate PopPK in patients with generalized anxiety disorder (CTR20231959), in order to develop a more scientific dosing regimen.

Cytochrome P450 enzymes (CYPs) play key roles in the metabolism of psychotropic drugs, especially CYP2C19, CYP2D6, and CYP3A4 (81). However, because of the high polymorphisms of CYPs, the therapeutic effects on patients are different, and approximately 30% of patients showing no response to SC (82). Thus, it is necessary to design PGx strategies in new drugs development to achieve personalized treatment. The current drug development and design strategies mainly focus on the population level, and personalized genomics research is relatively lacking and lagging behind (83). The FDA has added genomic labels for some psychotropic drugs and approved in 2020 that patients should test *CYP2C19* genotype when applying citalopram (75). It’s the first FDA-approved PGx-test for psychotropic drugs, indicating a breakthrough in PGx research on psychotropic drugs. Six clinical trials in our study were targeted to detect *CYP2D6* genetic polymorphisms, and the related drugs including SAL0114, SEP-363856, and aripiprazole. Besides, a phase II trial of TCM WenDanPian was aimed at *net T182C* locus polymorphisms. Those suggest that PGx is becoming a trend in drug development across various drug categories to ensure precision medication.

In addition to the above, some studies we displayed also employed biomarker detection and/or drug combination strategies. The determination of plasma biomarkers, especially inflammatory factors, provides a non-invasive way to explore the possible pathological changes and diagnostic indicators of psychiatric disorders (84–86). Currently, drug combination strategy is quite common in the treatment of cancer and chronic diseases. Considering the polygenic and complex etiology of psychiatric disorders, using drugs with multiple pharmacological effects or drug combinations with different molecular mechanisms may be a new approach to improve the therapeutic effect of psychotropic drugs (87).

### 4.4 Future directions

Psychiatric disorders have become a huge challenge threatening human health. The monoamine hypothesis, as the mainstream theory, is the basis for the development of a large number of drugs, especially antidepressants (88). However, the mere 30% effectiveness of antidepressants indicates that the monoamine hypothesis is not sufficient to explain the entire pathogenesis of psychiatric disorders (89). The increasing prevalence and limited progress have prompted a transformation in the direction of R&D for psychiatric disorders, namely the development of treatment strategies with novel mechanisms driven by new technologies. Our study has demonstrated a part of innovative targets, delivery systems, and design ideas involved in clinical trials conducted in China. Here we briefly overview the possible future R&D directions in this field worldwide, including emerging mechanism theories and upgraded delivery systems.

In addition to the traditional monoamine hypothesis, new theories proposed in recent decades mainly include the gene-environment interaction and the neuroplasticity. The increasing evidence suggests that the pathogenesis of psychiatric disorders is not only caused by individual gene mutations, but also the result of the interaction between genes and environmental risk factors, with clear biological characteristics (90). Thus, a new concept of gene-environment interaction was proposed, which may be mediated by epigenetics (91, 92). Epigenetic modifications play a crucial role in the human CNS physiology by regulating tissue-specific gene expression through DNA methylation, histone modification, and RNA interference (93, 94). Histone deacetylase (HDAC) is a key enzyme that maintains neuronal morphology and brain stability partly by catalyzing the deacetylation of histones (95). A series of inhibitor compounds such as RGFP-966 (HDAC3 inhibitor), T-518 (HDAC6 inhibitor), etc. have been proven to improve brain cognitive ability in AD mouse models (96–98). MeCP2 and ALKBH5 may affect the disease processes of AD and depression respectively by regulating methylation, which have been validated in animal models (99, 100).

Neuroplasticity refers to the ability of the brain to respond to internal and external stimuli through neurobiological changes (101). This theory overturns the traditional view of a “static brain” and believes that the nervous system undergoes lifelong dynamic changes. These changes can be adaptive and support the recovery of high-risk populations; otherwise, they may lead to neuropathology and psychiatric disorders (102). The AMPA receptor diffusional trapping machinery improves brain learning and memory function by regulating synaptic plasticity, making it a potential target for AD (103). Berberine reduces neuroinflammation by inhibiting the activation of NLRP3 inflammasome, thereby reversing neuroplastic damage and improved symptoms in depression model mice (104).

One of the reasons hindering the progress of psychiatric disorders treatment is related to delivery, and DDS technology innovation may significantly improve drug efficacy. The direction of future drug delivery mainly focuses on intelligent systems, including stimulus-responsive DDS and pulsatile DDS. Traditional delivery techniques suffer from non-specific biological distribution and uncontrollable release, leading to systemic side effects. Stimulus-responsive DDS can overcome the aforementioned issues. The design principle is to load drugs onto specific materials, and provide endogenous and exogenous stimuli (105). By changing or disrupting the carrier, drugs can be released at specific times and locations. Endogenous stimuli are triggered by the disease microenvironment, including pH, enzymes overexpressed at the lesion site, etc (106, 107); exogenous stimuli are external physical stimuli applied, such as light, temperature, ultrasound, etc (108, 109). By combining multiple stimuli, drug delivery efficiency and accuracy can be further improved (110). The hydrogel of Paliperidone (an antipsychotic drug) first enters the nasal cavity through intranasal route in the form of spray, and changes into a mucosal gel after exposure to physiological PH, thus playing a long-term controlled release role (94). Pulsatile DDS can release drugs based on preset time intervals or external signals (such as electricity, magnetism, ultrasound, etc.), making them suitable for drugs with intermittent dosing needs. Psychotropic drugs can use this system to enhance compliance (111).

### 4.5 Limitations

This study has certain limitations. First, according to the NMPA, clinical trials that meet one of the following criteria must be registered on the *chinadrugtrials.org.cn* platform prior to the start of the trial, and continuously updated on the progress: (1) approved by the NMPA and conducted in China; (2) approved for filing for bioequivalence testing of chemical drugs; and (3) phase IV clinical trials and post-marketing studies as required by Chinese regulations. However, there are still some trials that have not been included in this study due to not being required to register on this platform, such as trials in the early stages of development or investigator-initiated trials (IIT). Second, the platform did not provide detailed research stages and trial subjects information, so we were unable to conduct in-depth analysis and evaluation of treatment efficacy and adverse events. Third, due to the complex mechanisms of psychiatric disorders and the lack of detailed mechanism description, we did not further classify and analyze these drugs.

In conclusion, this study intuitively demonstrates the latest R&D progress of psychotropic drugs in China by analyzing the information of projects, indications, drugs, and trial design of PDCTs. It also introduced the research hotspots of psychotropic drugs from the perspectives of innovative drug target, drug optimization approach, and trial design strategies. Besides, we also introduced emerging mechanisms and delivery systems with promising applications. We are committed to providing insights into the latest stages of psychotropic drugs, delivering valuable information and references for the direction of psychotropic drugs development, the conduct of clinical trials, and individualized clinical dosing regimens.

## Data Availability

The original contributions presented in the study are included in the article/supplementary material. Further inquiries can be directed to the corresponding author.

## Glossary

R&D: research and development
PDCT: psychotropic drugs clinical trial
BE: bioequivalency
WHO: World Health Organization
ANX: anxiety
DEP: depression
CNS: central nervous system
BBB: blood-brain barrier
DR: dopamine receptor
5-HT: 5-hydroxytryptamine
GCP: Good Clinical Practice
DSM-V: Diagnostic and Statistical Manual of Mental Disorders, Fifth Edition
CDE: Center for Drug Evaluation
NMPA: National Medical Products Administration
NHC: National Health Commission
TCM: traditional Chinese medicine
SC: schizophrenia
PD: parkinsonism
AD: Alzheimer’s disease
BD: bipolar disorder
OCD: obsessive-compulsive disorder
WS: withdrawal symptoms
TS: tourette syndrome
MT1: melatonin MT1
MT2: melatonin MT2
TAAR1: trace amine-associated receptor 1
DRIP: dopamine receptor interacting protein
KOR: kappa-type opioid receptor
mAChR: muscarinic receptor
A2aR: adenosine A2a receptor
OX2R: orexin type 2 receptor
SLC6A3: solute carrier family 6 member 3
NE: norepinephrine
GABA_A_R: γ-aminobutyric acid A receptor subunit
NMDAR: N-methyl-D-aspartate receptor
QPCT: glutaminyl-peptide cyclotransferase
LRRK2: leucine rich repeat kinase 2
MAO-B: monoamine oxidase B
PDE 10: phosphodiesterase 10
GlyT1: glycine transporter 1
Aβ: amyloid β-protein
BDNF: brain derived neurotrophic factor
LYZ: lysozyme
SYP: synaptophysin
GPCR: G protein-coupled receptors
ICR: ion channel receptors
PopPK: population pharmacokinetics
PGx: pharmacogenomics
FDA: Food and Drug Administration
DDS: drug delivery system
HDAC: Histone deacetylase
